# Prognostic implications of conversion from nonshockable to shockable rhythms in out-of-hospital cardiac arrest

**DOI:** 10.1186/s13054-014-0528-7

**Published:** 2014-09-22

**Authors:** Yoshikazu Goto, Tetsuo Maeda, Yumiko Nakatsu-Goto

**Affiliations:** Section of Emergency Medicine, Kanazawa University Hospital, 13-1 Takaramachi, Kanazawa, 920-8641 Japan; Department of Cardiology, Yawata Medical Center, 12-7 I Yawata, Komatsu, 923-8551 Japan

## Abstract

**Introduction:**

The prognostic significance of conversion from nonshockable to shockable rhythms in patients with initial nonshockable rhythms who experience out-of-hospital cardiac arrest (OHCA) remains unclear. We hypothesized that the neurological outcomes in those patients would improve with subsequent shock delivery following conversion to shockable rhythms and that the time from initiation of cardiopulmonary resuscitation by emergency medical services personnel to the first defibrillation (shock delivery time) would influence those outcomes.

**Methods:**

We analyzed the data of 569,937 OHCA adults with initial nonshockable rhythms. The data were collected in a nationwide Utstein-style Japanese database between 2005 and 2010. Patients were divided into subsequently shocked (*n* =21,944) and subsequently not-shocked (*n* =547,993) cohorts. The primary study endpoint was 1-month favorable neurological outcome (Cerebral Performance Categories scale, category 1 or 2).

**Results:**

In the subsequently shocked cohort, the ratio of 1-month favorable neurological outcome was significantly higher than that in the subsequently not-shocked cohort (1.79% versus 0.60%, *P* <0.001). Multivariate logistic regression analysis for 11 prehospital variables revealed that when the shock delivery time was less than 20 minutes, subsequent shock delivery was significantly associated with increased odds of 1-month favorable neurological outcomes (adjusted odds ratios (95% confidence interval), 6.55 (5.21 to 8.22) and 2.97 (2.58 to 3.43) for shock delivery times less than 10 minutes and from 10 to 19 minutes, respectively). However, when the shock delivery time was more than or equal to 20 minutes, subsequent shock delivery was not associated with increased odds of 1-month favorable neurological outcomes.

**Conclusions:**

In patients with an initial nonshockable rhythm after OHCA, subsequent conversion to shockable rhythms during emergency medical services resuscitation efforts was associated with increased odds of 1-month favorable neurological outcomes when the shock delivery time was less than 20 minutes.

## Introduction

Despite important advances in preventive measures, cardiac arrest remains a substantial public health problem and a leading cause of death in many parts of the world [1]. Adult patients who experience out-of-hospital cardiac arrest (OHCA) typically experience sudden, unexpected ventricular fibrillation (VF) and often have underlying coronary artery disease with myocardial ischemia [2]. Outcomes for patients with initial shockable rhythms (VF and pulseless ventricular tachycardia) are often excellent, but those for patients with nonshockable initial rhythms (pulseless electrical activity (PEA) and asystole) are generally poor [2-6].

During the past two decades, the incidence of initial shockable rhythms after cardiac arrest has declined significantly and a complementary increase in nonshockable initial rhythms has been observed by emergency medical services (EMS) personnel who treat OHCA [3,5,6]. In recent population-based studies, 76.3% to 92.6% of OHCA patients presented with an initial nonshockable rhythm [3,5,7,8].

Although defibrillation for shockable rhythms has received strong emphasis in the recent guidelines for cardiopulmonary resuscitation (CPR) [1], it remains controversial whether defibrillation of a shockable rhythm that followed an initial nonshockable rhythm is associated with an improved outcome in OHCA patients [9-14]. In 2007, Hallstrom *et al*. noted a low odds ratio (OR) of 0.18 (adjusted OR; *P* =0.036) for survival to hospital discharge in OHCA patients with subsequently shockable rhythms relative to those who did not convert to shockable rhythms [10]. However, in three earlier studies of OHCA, researchers reported that defibrillation of a subsequently shockable rhythm was associated with improved outcomes compared with the outcomes in patients with initial nonshockable rhythms who did not convert to a shockable rhythm [11-13]. Moreover, Thomas *et al*. recently reported that survival to hospital discharge for OHCA patients with an initial nonshockable rhythm was not associated with conversion to a shockable rhythm during EMS resuscitation efforts (adjusted OR, 0.88; 95% confidence interval (CI), 0.60 to 1.30) [14]. It should be noted that there were differences between the EMS systems in these studies; however, the crucial point is that the time from the initiation of CPR by EMS personnel to the first defibrillation (shock delivery time) was not considered as a confounding factor in the analyses. Therefore, our first objective in the present study was to examine whether neurological outcomes in patients with OHCA who had an initial nonshockable rhythm would improve with subsequent conversion to shockable rhythm following defibrillation. Our second objective was to determine whether the shock delivery time would be associated with 1-month neurological outcomes.

## Materials and methods

### Study design and data source

The present investigation was a nationwide, population-based observational study of all adult patients (ages ≥18 years) for whom resuscitation had been attempted after OHCA in Japan between 1 January 2005 and 31 December 2010. Cardiac arrest was defined as the cessation of cardiac mechanical activity as confirmed by the absence of signs of circulation [3]. The cause of arrest was presumed to be cardiac unless evidence suggested external causes (trauma, hanging, drowning, drug overdose and asphyxia), respiratory diseases, cerebrovascular diseases, malignant tumors or any other noncardiac cause. The determination of the cause as noncardiac or cardiac was made by the physicians in charge in collaboration with the EMS personnel. This study was approved by the ethics committee of Kanazawa University. According to the informed consent guidelines in Japan [15], it is unnecessary to obtain informed consent from each patient to use secondary data such as those contained in this anonymous database. Therefore, the requirement for written informed consent was waived.

### EMS system in Japan

Japan has approximately 127 million residents in an area of 378,000 km^2^, approximately two-thirds of which is uninhabited mountainous terrain [16]. Details of the Japanese EMS system have been described previously [17]. Briefly, municipal governments provide EMS through approximately 800 fire stations with dispatch centers. The Fire and Disaster Management Agency (FDMA) of Japan supervises the nationwide EMS system, whereas each local EMS system is operated by the local fire station. Generally, an ambulance crew includes three EMS staff, including at least one emergency lifesaving technician (ELST). ELSTs are allowed to use several resuscitation methods, including semiautomated external defibrillators, insertion of a supraglottic airway device, insertion of a peripheral intravenous line and administration of Ringer’s lactate solution. Since July 2004, only specially trained ELSTs have been permitted to insert a tracheal tube, and, since April 2006, they have been permitted to administer intravenous epinephrine in the field under the instruction of an online physician. Since October 2006, all EMS providers perform CPR according to the Japanese CPR guidelines [18], which are based on the 2005 American Heart Association guidelines [19]. As EMS personnel in Japan are legally prohibited from terminating resuscitation in the field, most OHCA patients receive CPR from EMS providers and are transported to hospitals, except in cases where fatality is certain [20,21]. The length of the on-scene effort by EMS personnel is not predetermined before transport is initiated.

### Data collection and quality control

The FDMA launched a prospective, population-based, observational study including all OHCA patients who received EMS in Japan since January 2005 [17]. EMS personnel at each center recorded the data for OHCA patients with the cooperation of the physician in charge, using an Utstein-style template [22]. All data were stored in the nationwide database developed by the FDMA for public use. The data were transferred to the individual fire stations and subsequently integrated into the registry system on the FDMA database server. The data were checked for consistency by the computer system and confirmed by the FDMA. If the data form was incomplete, the FDMA returned it to the respective fire station and the form was completed. All data were transferred and stored in the nationwide database developed by the FDMA for public use [3]. The FDMA gave us permission to analyze this database and provided all the anonymous data to our research group. The main items included in the data set were as follows: sex, age, cause of arrest (presumed cardiac origin or not), bystander witness status, bystander CPR with or without automated external defibrillator use, initial identified cardiac rhythm, bystander category (that is, the presence or absence of a bystander or whether the bystander was a layperson or an EMS professional), whether epinephrine was administered, whether advanced airway management techniques (including endotracheal tube, laryngeal mask airway and esophageal-tracheal tube) were used, whether return of spontaneous circulation (ROSC) was achieved before arrival at the hospital, time of the emergency call, time of vehicle arrival at the scene, time of initiation of CPR by EMS personnel, time of ROSC, time of vehicle arrival at the hospital, time of epinephrine administration, time of shock delivery by EMS personnel, 1-month survival and neurological outcome at 1 month after cardiac arrest. Several resuscitation methods that EMS personnel used were recorded on a recording medium for the EMS reports as a written record or as an audio recording. The time data were recorded electronically on a record medium according to the times on the clock used by the EMS system that responded to the call [3]. Especially, the time of first shock delivery was validated using the data from defibrillator recordings. The neurological outcome was defined using the Cerebral Performance Categories (CPC) scale: category 1, good cerebral performance; category 2, moderate cerebral disability; category 3, severe cerebral disability; category 4, coma or vegetative state; and category 5, death [22]. The CPC categorization was determined by the physician in charge.

### Endpoints

The primary study endpoint was 1-month favorable neurological outcome (defined as a CPC score of 1 or 2) [22]. The secondary endpoints were prehospital ROSC and survival at 1 month after the OHCA.

### Statistical analysis

Kolmogorov–Smirnov–Lilliefors tests were performed to evaluate the distributions of continuous variables, and we found that all continuous variables had a nonnormal distributions (all *P* <0.01). Therefore, the Kruskal–Wallis test for continuous variables and the χ^2^ test for categorical variables were performed to compare the characteristics or outcomes between the cohorts. Multivariate logistic regression analyses including 11 variables were performed to assess the factors associated with increased odds of prehospital ROSC, 1-month survival and 1-month CPC score of 1 or 2 for all eligible patients. These data included year, age, sex, arrest witnessed by any layperson, arrest witnessed by EMS personnel, bystander CPR, presumed cause of arrest, initial cardiac rhythm, subsequent shock delivery, call-to-response time and prehospital epinephrine administration as independent variables. The analytical models yielded concordance statistics of 0.81 for prehospital ROSC, 0.75 for 1-month survival and 0.84 for 1-month CPC score of 1 or 2, which indicated good discrimination.

The call-to-response time was calculated as the time from the emergency call to the time of vehicle arrival at the scene. We defined shock delivery time as the time interval from the initiation of CPR by EMS personnel to the first defibrillation. To associate shock delivery time with whether shock was received, we classified prehospital shock delivery variables into five categories in increments of 10 minutes, referring to median values of shock delivery time: No, Yes (<10 minutes), Yes (10 to 19 minutes), Yes (20 to 29 minutes) and Yes (≥30 minutes), where the figures in parentheses are the shock delivery times. We also defined epinephrine administration time as the time interval from the initiation of CPR by EMS personnel to the first epinephrine administration. To associate the epinephrine administration time with whether epinephrine was received, we classified prehospital epinephrine administration variables into four categories in increments of 10 minutes, referring to median value of epinephrine administration time: No, Yes (<10 minutes), Yes (10 to 19 minutes) and Yes (≥20 minutes), where the figures in parentheses are the epinephrine administration times.

Continuous variables are expressed as medians with 25th to 75th percentiles. Categorical variables are expressed as percentages. As estimates of effect size and variability, we report ORs with 95% CIs. All statistical analyses were performed using the JMP statistical package version 10 (SAS Institute, Cary, NC, USA). All tests were two-tailed, and a value of *P* <0.05 was considered statistically significant.

## Results

**Figure 1:**
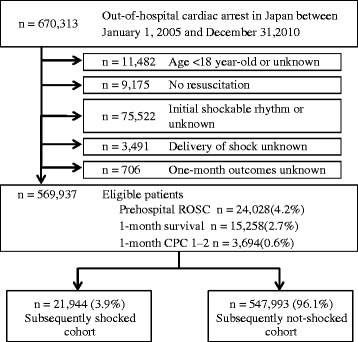
**Study profile with the selection of participants.** CPC, Cerebral Performance Categories scale; ROSC, Return of spontaneous circulation.

During the 6-year study period, 670,313 patients were documented in the database. We excluded patients with initial shockable rhythms (VF and pulseless ventricular tachycardia) and finally considered 569,937 (85.0%) patients with initial nonshockable rhythms (PEA and asystole) eligible for enrollment into this study. Figure 1 shows a flow diagram depicting the inclusion and exclusion criteria for patients in the present study. The overall prehospital ROSC, 1-month survival and 1-month favorable neurological outcomes (CPC scores 1 and 2) rates were 4.2% (*n* =24,028), 2.7% (*n* =15,258) and 0.6% (*n* =3,694), respectively. Patients were divided into two cohorts: subsequently shocked (*n* =21,944) or subsequently not shocked (*n* =547,993). Those patients who converted to shockable rhythms were identified by shocks later in the course of resuscitation and were assigned to the subsequently shocked cohort. The delivery of shocks was used as a surrogate maker for conversion to a shockable rhythm. Conversely, the subsequently not-shocked cohort was composed of those who received no subsequent shocks during their resuscitation.

Table 1 shows the baseline characteristics and the results of the analyses of the two cohorts. Because of the large size of the study population, several significant differences were noted in baseline characteristics between the two cohorts; however, sizeable differences were less frequent, except for the ratios of witnessed arrest, initial cardiac rhythm and epinephrine administration. The subsequently shocked cohort had significantly higher prehospital ROSC, 1-month survival and 1-month CPC score of 1 or 2 than the subsequently not-shocked cohort (all *P* <0.0001).

**Table 1:** **Baseline characteristics of the study cohorts according to subsequent shock delivery** ^**a**^

**Characteristics**	**All patients with initial nonshockable rhythm,** ***n*** **(%)**		**Subsequently shocked cohort,** ***n*** **(%)**		**Subsequently not-shocked cohort,** ***n*** **(%)**		***P*** **-value**
Total patients in each group (%)	569,937	(100)	21,944	(3.9)	547,993	(96.1)	
Year							
2005	87,978	(15.4)	3,978	(18.1)	84,000	(15.3)	<0.0001
2006	90,942	(16.0)	3,618	(16.5)	87,324	(15.9)	
2007	88,710	(15.6)	3,465	(15.8)	85,245	(15.6)	
2008	97,211	(17.1)	3,504	(16.0)	93,707	(17.1)	
2009	99,024	(17.3)	3,680	(16.8)	95,344	(17.4)	
2010	106,072	(18.6)	3,699	(16.9)	102,373	(18.7)	
Age, yr	77	(65 to 85)	75	(62 to 83)	77	(66 to 85)	<0.0001
Male	324,683	(57.0)	13,819	(63.0)	310,864	(56.7)	<0.0001
Witnessed OHCA	204,687	(35.9)	10,595	(48.3)	194,092	(35.4)	<0.0001
Witnessed OHCA by EMS personnel	30,341	(5.3)	1,226	(5.6)	29,115	(5.3)	0.076
Bystander CPR	224,273	(39.4)	8,842	(40.3)	215,431	(39.3)	0.0035
Presumed cardiac cause	303,947	(53.3)	14,357	(65.4)	289,590	(52.9)	<0.0001
Initial cardiac rhythm							
Pulseless electrical activity	138,044	(24.2)	8,008	(36.5)	130,036	(23.7)	<0.0001
Asystole	431,893	(75.8)	13,936	(63.5)	417,957	(76.3)	
Call-to-response time, min	7.0	(5 to 9)	7.0	(5 to 9)	7.0	(5 to 9)	<0.0001
Time from the initiation of CPR by EMS personnel to hospital arrival, min	30	(24 to 37)	32	(26 to 39)	30	(24 to 36)	<0.0001
Shock delivery time, min^b^	20.0	(15 to 27)	20.0	(15 to 27)	No data		
Epinephrine administration	33,772	(5.9)	2,883	(13.1)	30,889	(5.6)	<0.0001
Epinephrine administration time, min^c^	15	(10 to 20)	14	(10 to 20)	15	(10 to 20)	0.006
Outcomes							
Prehospital ROSC	24,028	(4.2)	1,676	(7.6)	22,352	(4.1)	<0.0001
1-month survival	15,258	(2.7)	1,055	(4.8)	14,203	(2.6)	<0.0001
1-month favorable neurological outcome (CPC category 1 or 2)	3,694	(0.6)	393	(1.8)	3,301	(0.6)	<0.0001

^a^CPC, Cerebral Performance Categories scale; CPR, Cardiopulmonary resuscitation; EMS, Emergency medical services; OHCA, Out-of-hospital cardiac arrest; ROSC, Return of spontaneous circulation. ^b^Time from the initiation of CPR by EMS personnel to the first shock delivery. ^c^Time from the initiation of CPR by EMS personnel to the first epinephrine administration. Values are reported either as either number of patients (%) or median (25th to 75th percentiles). Values were missing for 355 to 422 individuals across time variables.

Table 2 shows the results of multivariate logistic regression analyses including 11 variables to determine the factors associated with prehospital ROSC, 1-month survival, and 1-month CPC 1–2. When the shock delivery time was <20 minutes, it was significantly associated with increased odds of prehospital ROSC (adjusted OR [95% CI], 4.06 [3.47–4.75] and 1.73 [1.59–1.88]; for shock delivery times of <10 minutes and 10–19 minutes, respectively), 1-month survival (adjusted OR [95% CI], 4.7 [3.99–5.53] and 2.16 [1.98–2.37]; for shock delivery times of <10 minutes and 10–19 minutes, respectively), and 1-month CPC 1–2 (adjusted OR [95% CI], 6.55 [5.21–8.22] and 2.97 [2.58–3.43]; for shock delivery times of <10 minutes and 10–19 minutes, respectively).

**Table 2:** **Results of multivariate logistic regression analyses for variables associated with outcomes** ^**a**^

**Variables**	**Adjusted OR (95% CI)**					
	**Prehospital ROSC**		**1-month survival**		**1-month CPC category 1 or 2**	
Year						
2005	Reference		Reference		Reference	
2006	1.04	(0.99 to 1.10)	1.04	(0.98 to 1.10)	0.99	(0.89 to 1.12)
2007	1.04	(0.99 to 1.10)	1.06	(0.99 to 1.12)	1.24	(1.10 to 1.40)
2008	1.04	(0.99 to 1.10)	1.01	(0.96 to 1.08)	1.27	(1.12 to 1.43)
2009	1.08	(1.03 to 1.13)	1.07	(1.01 to 1.14)	1.49	(1.32 to 1.68)
2010	1.13	(1.08 to 1.19)	1.15	(1.08 to 1.21)	1.55	(1.38 to 1.75)
Age^b^	0.99	(0.99 to 0.99)	0.99	(0.99 to 0.99)	0.98	(0.98 to 0.98)
Male	0.92	(0.89 to 0.94)	0.94	(0.91 to 0.97)	0.93	(0.92 to 0.94)
Witnessed OHCA	2.24	(2.17 to 2.31)	2.27	(2.18 to 2.35)	2.76	(2.54 to 3.01)
Witnessed OHCA by EMS personnel	1.18	(1.13 to 1.24)	1.40	(1.33 to 1.48)	2.11	(1.94 to 2.30)
Bystander CPR	1.09	(1.06 to 1.13)	0.99	(0.96 to 1.03)	0.88	(0.81 to 0.95)
Presumed cardiac cause	0.56	(0.55 to 0.58)	0.71	(0.69 to 0.74)	1.28	(1.20 to 1.37)
Initial cardiac rhythm						
Pulseless electrical activity	3.58	(3.48 to 3.69)	3.06	(2.95 to 3.17)	5.18	(4.78 to 5.61)
Asystole	Reference		Reference		Reference	
Call-to-response time^b^	1.00	(0.99 to 1.00)	1.00	(1.00 to 1.00)	0.93	(0.92 to 0.94)
Shock delivery^c^						
No	Reference		Reference		Reference	
Yes (<10 min)	4.06	(3.47 to 4.75)	4.7	(3.99 to 5.53)	6.55	(5.21 to 8.22)
Yes (10 to 19 min)	1.73	(1.59 to 1.88)	2.16	(1.98 to 2.37)	2.97	(2.58 to 3.43)
Yes (20 to 29 min)	0.92	(0.83 to 1.03)	0.87	(0.76 to 1.01)	0.97	(0.73 to 1.27)
Yes (≥30 min)	1.02	(0.90 to 1.16)	0.77	(0.63 to 0.95)	0.82	(0.53 to 1.25)
Epinephrine administration^d^						
No	Reference		Reference		Reference	
Yes (<10 min)	7.44	(6.97 to 7.93)	1.71	(1.53 to 1.91)	0.85	(0.64 to 1.11)
Yes (10 to 19 min)	5.53	(5.29 to 5.78)	1.23	(1.14 to 1.33)	0.48	(0.36 to 0.65)
Yes (≥20 min)	3.73	(3.50 to 3.98)	0.74	(0.65 to 0.84)	0.49	(0.40 to 0.61)

^a^CI, Confidence interval; CPC, Cerebral Performance Categories scale; CPR, Cardiopulmonary resuscitation; EMS, Emergency medical services; OHCA, Out-of-hospital cardiac arrest; OR, Odds ratio; ROSC, Return of spontaneous circulation. ^b^Adjusted odds ratios are reported for unit odds. ^c^If shock was received, variables were divided into four categories according to the time from the initiation of CPR by EMS personnel to the first shock delivery (shock delivery time). ^d^If prehospital epinephrine was received, variables were divided into three categories according to the time from the initiation of CPR by EMS personnel to the first epinephrine administration (epinephrine administration time).

Table 3 shows the results of subgroup analyses for 1-month outcomes in the subsequently shocked delivery cohort. PEA was significantly associated with increased adjusted ORs for 1-month survival and 1-month favorable neurological outcomes compared with asystole. Epinephrine administration was significantly associated with decreased adjusted OR for 1-month favorable neurological outcomes.

**Table 3:** **Subgroup analyses for 1-month outcomes in subsequently shocked cohort** ^**a**^

**Subsequently shocked cohort characteristics (** ***N*** **=21,944)**	**1-month survival**				**1-month CPC category 1 or 2**			
	***n***	**Rate (%)**	**Crude OR (95% CI)**	**Adjusted OR** ^**b**^ **(95% CI)**	***n***	**Rate (%)**	**Crude OR (95% CI)**	**Adjusted OR** ^**b**^ **(95% CI)**
Initial cardiac rhythm								
Asystole, *n* =13,936, 63.5%	499	3.58	Reference	Reference	143	1.03	Reference	Reference
PEA, *n* =8,008, 36.5%	556	6.94	2.01 (1.77 to 2.28)	1.61 (1.41 to 1.83)	250	3.12	3.11 (2.53 to 3.83)	2.24 (1.80 to 2.79)
Epinephrine administration								
No, *n* =19,061, 86.9%	914	4.80	Reference	Reference	359	1.88	Reference	Reference
Yes, *n* =2,883, 13.1%	141	4.89	1.02 (0.85 to 1.22)	0.84 (0.68 to 1.01)	34	1.18	0.62 (0.43 to 0.87)	0.43 (0.29 to 0.61)

^a^CI, Confidence interval; CPC, Cerebral Performance Categories scale; OR, Odds ratio; PEA, Pulseless electrical activity. ^b^Adjustment for potential confounders included ten variables: years, age, sex, witnessed arrest, witnessed arrest by emergency medical services personnel, bystander cardiopulmonary resuscitation, presumed cardiac etiology, initial cardiac rhythm, call-to-response time and epinephrine administration.

## Discussion

The present analysis of approximately 570,000 adult patients with an initial nonshockable rhythm after OHCA in Japan demonstrates that the crude ratios of prehospital ROSC, 1-month survival and 1-month favorable neurological outcomes in the subsequently shocked cohort were significantly higher than those in the subsequently not-shocked cohort. Multivariate logistic regression analyses revealed that subsequent shock delivery in patients with an initial nonshockable rhythm was significantly associated with increased odds of prehospital ROSC, 1-month survival and 1-month favorable neurological outcomes when the shock delivery time was less than 20 minutes.

Table 4 shows a comparison of the adjusted ORs of subsequent shock delivery for improving outcomes in five previous reports on the subject. Hallstrom *et al*. [10] showed that subsequent shock delivery with a mean shock delivery time of 21.0 minutes was associated with a decreased OR for survival to hospital discharge. Kajino *et al*. [12], however, reported contrary findings. They found that subsequent shock delivery with a mean shock delivery time of 12.3 minutes was associated with an increased OR for a 1-month CPC 1 or 2. Although these two findings seem to be conflicting, when the shock delivery time is considered, the results of the present study support those in both the Hallstrom and Kajino studies. We found that when the shock delivery time is less than 20 minutes, subsequent shock delivery may be associated with increased ORs for 1-month survival and 1-month CPC 1 or 2. However, when the shock delivery time is 20 minutes or longer, subsequent shock delivery may be associated with decreased ORs for 1-month survival and 1-month CPC 1 or 2. In other words, the relationship between outcomes after OHCA and subsequent shock delivery after an initial nonshockable rhythm may be substantially associated with shock delivery time. Herlitz *et al*. [11] and Olasveengan *et al*. [13] also found that the need for defibrillation was associated with an improved outcome. Herlitz *et al*. speculated that some of the patients who were judged to have a nonshockable rhythm actually had a fine VF, indicating the possibility of successful defibrillation. Olasveengan *et al*. pointed out that more pauses in chest compression in the subsequent shockable cohort might be of limited clinical importance compared with defibrillation attempts. However, these two positive studies of subsequent shock delivery [11,13] did not indicate the shock delivery time and did not discuss its relationship with outcomes. Thomas *et al*. [14] recently demonstrated that conversion to a shockable rhythm was not associated with improved survival. They hypothesized that their findings might simply have been due to differences in the etiologies of the nonshockable rhythm. It is plausible that some nonshockable arrest etiologies may actually benefit from subsequent shock delivery, whereas others may not. We could not precisely determine the etiology of cardiac arrest in the nonshockable patients in the present study, because the relevant data were insufficient. From the viewpoint of shock delivery time, the results of the Thomas *et al*. study are consistent with those from the patients in our study who had shock delivery times of 20 to 29 minutes and showed an adjusted OR of 0.87 (95% CI, 0.76 to 1.01) for 1-month survival after OHCA (Table 2). Therefore, a potential explanation for the findings of Thomas *et al*. may be the shock delivery time, although precise data concerning shock delivery times were not provided in that study.

**Table 4:** **Comparison of adjusted odds ratios of subsequent shock delivery for improving outcomes in previous studies** ^**a**^

**Study**	**Year of publication**	***n***	**Endpoint**	**Proportion of patients with conversion to shockable rhythm**	**Shock delivery time** ^**b**^ **, mean (SD), min**	**Adjusted OR**	**95% CI**
Hallstrom *et al*. [10]	2007	738	Survival to hospital discharge	22.2%	21.0 (8.1)^c^	0.18	Unknown (*P* =0.036)
Herlitz *et al*. [11]	2008	22,465	1-month survival	26%	Unknown	1.96	1.49 to 2.56
Kajino *et al*. [12]	2008	12,353	1-month CPC 1 or 2	4.8%	12.3 (6.9)	4.3	2.8 to 6.7
Olasveengan *et al*. [13]	2009	751	Survival to hospital discharge	13.0%	Unknown	3.02	1.07 to 8.57
Thomas *et al*. [14]	2013	6,556	Survival to hospital discharge	18.9%	Unknown	0.89	0.55 to 1.45

^a^CI, Confidence interval; CPC, Cerebral Performance Categories scale; OR, Odds ratio; SD, Standard deviation. ^b^Time from the initiation of cardiopulmonary resuscitation by emergency medical services personnel to the first defibrillation. ^c^Numbers were calculated using data from the original papers.

Table 4 also shows the proportions of patients with conversion to a shockable rhythm in five previous studies. There is considerable difference in the proportion that transitioned to receive a shock across these studies. In the present study, only 3.9% of patients with nonshockable rhythms subsequently received shocks (Table 1). This low proportion is similar to the published Osaka experience (4.8%) [12]. In contrast, four other studies indicated a much larger proportion of patients (from 13.0% to 26%). One of the reasons for this difference may be due to the EMS systems in Japan. As EMS personnel in Japan are not allowed to perform termination of resuscitation in the field, most OHCA patients are transported to hospitals. Therefore, some patients who would not be transported to hospitals in other systems outside Japan may be counted for calculating the proportion of subsequently shocked patients. This difference in the proportion of patients with conversion to a shockable rhythm may have influenced our present results as a confounding factor.

According to the “three-phase model” [23], which includes an electrical phase, a circulatory phase and a metabolic phase of CPR to reflect the time-sensitive progression of resuscitation physiology, the optimal treatment of cardiac arrest requires distinct, phase-specific initial therapy to improve the survival of patients with an initial VF rhythm. In the metabolic phase (approximately 10 minutes or more after cardiac arrest), the effectiveness of both immediate defibrillation and CPR, followed by defibrillation, decreases rapidly and survival rates appear to be poor. Moreover, Reynolds *et al*. [24] recently showed that CPR was most effective within the first 10 to 15 minutes and that the probability of favorable neurological recovery fell to 2% beyond this point. Adjusting for both prehospital and inpatient covariates, they demonstrated that CPR duration (minutes) was independently associated with a decreased OR for a favorable functional status at hospital discharge (adjusted OR, 0.84; 95% CI, 0.72 to 0.98). In the present study, the median shock delivery time was 20 minutes (25th to 75th percentiles, 15 to 27) in the subsequently shocked cohort. This means that almost all patients with a subsequent shockable rhythm were in the metabolic phase of the three-phase model. Considering the importance of time in resuscitation, patients with a subsequent shockable rhythm may require different treatment strategies, depending on the time of rhythm conversion from the initial nonshockable rhythm. Namely, if the patient with an initial nonshockable rhythm has a rhythm conversion to shockable rhythm within 20 minutes from the initiation of CPR by EMS personnel, a shock might be delivered; if the patient has a sustained nonshockable rhythm beyond 20 minutes after the initiation of CPR by EMS personnel, a shock might not be delivered, and high-quality CPR could be performed with minimal interruption, appropriate ventilation and identification and treatment of reversible causes.

Eilevstjønn *et al*. [25] demonstrated that cardiac rhythms before shock delivery were related to outcomes (ROSC). They found that prior PEA before shock was superior, with an OR of 2.4 (95% CI, 1.2 to 4.8), and that prior asystole was inferior, with an OR of 0.15 (95% CI, 0.05 to 0.51), compared to initial VF (reference) with regard to the probability of ROSC. In the present study, prior PEA before shock was significantly associated with an increased adjusted OR for 1-month favorable neurological outcomes compared with asystole (Table 3). Thus, the results of the present study support those reported by Eilevstjønn *et al*. Moreover, Nordseth *et al*. [26] recently indicated that the optimal first-loop duration of chest compressions may be 4 minutes in patients with initial PEA and 6 to 8 minutes in patients with initial asystole. Accordingly, a new paradigm may be required, especially in patients with initial PEA as opposed to those with initial asystole, to achieve more than modest improvements in patients with an initial nonshockable rhythm.

In our present study, we also demonstrate that those patients who converted to a shockable rhythm with administration of epinephrine did not improve, whereas those who converted to a shockable rhythm without use of epinephrine had a relatively favorable neurological outcome after shock delivery (Table 3). The β-effects of epinephrine on the heart, which are not beneficial during or after cardiac arrest, may worsen myocardial dysfunction and increase myocardial oxygen consumption [27]. These adverse effects of epinephrine may contribute substantially to the poor prognosis in the subsequently shockable patients with OHCA.

Hall *et al*. [28] evaluated the association between the physiology of the heart and outcomes in patients with initial (primary group) and subsequent (secondary group) VF with OHCA by analyzing the quantitative waveform of VF. They found that waveform measures of VF for the secondary group were quite similar to those for the primary group. However, the survival rate was 37% in the restricted, matched primary group and 0% in the secondary group. These findings suggest that, although resuscitation is successful in some secondary VF cases, brain injury or systemic dysfunction could contribute substantially to mortality. During the metabolic phase of the three-phase model, tissue injury due to global ischemic events and to reperfusion can result in circulating metabolic factors that cause additional injury beyond the effects of local ischemia [23]. Conceivably, global whole-body ischemia and cellular reperfusion injury may be more critical in the subsequently shockable patients than in the initial shockable patients.

### Study limitations

The potential limitations of the present analysis are as follows. First, our database lacked the detailed data needed to permit further risk adjustment for outcomes, such as for comorbid diseases of patients, location where the OHCA occurred, quality of EMS personnel, the degree of regional differences among EMS centers, in-hospital medication (for example, additional shock delivery, additional vasopressor (epinephrine and/or vasopressin) use and percutaneous coronary intervention) and the availability of specialists in emergency care (cardiologists). These deficient data were associated with our retrospective record review study design. Second, we cannot exclude the possibility of uncontrolled confounders, even though we used a uniform data collection procedure based on the Utstein-style guidelines for reporting cardiac arrest and had a large sample size and population-based study design. Third, as with all epidemiological studies, the integrity, validity and ascertainment bias of the data were potential limitations. Fourth, we could not exclude patients with shock for a wrong indication due to electrical misreading. Fifth, we should stress that caution must be exercised when generalizing these results to other EMS systems.

## Conclusions

We found that patients with initial nonshockable rhythms after OHCA could develop a shockable rhythm later on in the resuscitation, and some of these patients had favorable outcomes. Notably, subsequent conversion to shockable rhythms during EMS resuscitation efforts was associated with increased odds of prehospital ROSC, 1-month survival and 1-month favorable neurological outcomes when the shock delivery time was less than 20 minutes.

## Key messages

- We analyzed the data collected over 6 years for 569,937 OHCA patients with initial nonshockable rhythms using a nationwide Utstein-style Japanese database.
- We determined that the ratios of prehospital ROSC, 1-month survival and 1-month favorable neurological outcomes in the subsequently shocked cohort were significantly higher than those in the subsequently not-shocked cohort (7.64% versus 4.08%, 4.81% versus 2.59% and 1.79% versus 0.60%, respectively; all *P* <0.001).
- Multivariate logistic regression analyses for 11 prehospital variables revealed that subsequent shock delivery for OHCA patients with initial nonshockable rhythms was independently associated with increased odds of prehospital ROSC, 1-month survival and 1-month favorable neurological outcome when the shock delivery time was less than 20 minutes.

## Abbreviations

CI: Confidence interval
CPC: Cerebral Performance Categories
CPR: Cardiopulmonary resuscitation
ELST: Emergency lifesaving technician
EMS: Emergency medical services
FDMA: Fire and Disaster Management Agency
OHCA: Out-of-hospital cardiac arrest
OR: Odds ratio
PEA: Pulseless electrical activity
ROSC: Return of spontaneous circulation
VF: Ventricular fibrillation
